# Validity of a novel optical coherence tomography angiography flow index in a cohort of primary open angle glaucoma

**DOI:** 10.1186/s12886-023-03108-8

**Published:** 2023-09-05

**Authors:** Ahmed Ameen Ismail, Sherin Sadek, Mahmoud Kamal, Ragai Hatata

**Affiliations:** Department of Ophthalmology, Faculty of Medicine, Fayoum University, Al Fayoum, Egypt

**Keywords:** Optical coherence tomography angiography, Mathematical model, Flow index, Glaucoma, Hagen-Poiseuille law

## Abstract

**Background:**

Vascular mechanisms are implicated in many ocular diseases. Therefore, different vascular imaging modalities are used in management of such conditions. Optical coherence tomography angiography (OCTA) has high spatial resolution and segmentable 3D volumetric sampling enabling isolation of retinal and peripapillary vascular beds. However, OCTA only indirectly derives quantitative flow data i.e. velocimetry through methods and algorithms liable to limitations like signal saturation. This study introduces and validates novel mathematical OCTA flow indices that may compensate for some OCTA velocimetric limitations.

**Methods:**

Thirty-seven eyes of 23 POAG patients were included. Each underwent baseline and follow-up assessment one month thereafter. Assessment comprised full ophthalmological examination, intraocular pressure (IOP), systemic arterial blood pressure (SABP) and OCTA macula and ONH. Angiograms were processed using ImageJ to calculate *OCTA intensity-based flow indices (FIOs)*, for superficial vascular plexus (SVP), deep vascular plexus (DVP) and optic nerve head vascular plexus (ONH-RPC), i.e. SFIO, DFIO and ONHFIO respectively. Mean ocular perfusion pressure (MOPP) was calculated using IOP and SABP. OCTA vascular densities (VD) and MOPP were used to calculate three respective *mathematical flow indices (FIMs)* for SVP, DVP and ONH-RPC, based on Hagen-Poiseuille law, i.e. SFIM, DFIM, ONHFIM respectively. Pearson test was used for correlation between the two sets of indices. Intraclass correlation coefficient (ICC) was tested for baseline and follow-up values for each index.

**Results:**

There was positive correlation between the three FIMs and their respective FIOs at baseline and follow-up ranging between high and moderate. Correlation coefficients (CCs) were 0.773 and 0.609 for SFIM and SFIO *P*-value < 0.001, 0.829 and 0.624 for DFIM and DFIO *P*-value < 0.001 and 0.516 and 0.737 for ONHFIM *P*-value = 0.001 for baseline and follow-up respectively. ICCs were 0.772 *P*-value < 0.001, 0.328 *P*-value = 0.022 and 0.888 *P*-value < 0.001 for SFIM, DFIM and ONHFIM respectively. For SFIO, DFIO and ONHFIO, ICCs were 0.420 *P*-value = 0.004, 0.079 *P*-value = 0.320 and 0.833 *P*-value < 0.001 respectively.

**Conclusion:**

The novel FIMs are reliable alternatives to FIOs and may compensate for OCTA signal saturation in extremes of MOPP. SFIM and ONHFIM showed high ICCs with excellent reliability. While DFIM demonstrated low ICC indicating poor reliability, it still performed better than its corresponding DFIO.

## Background

Myriad ocular diseases have a primarily vascular pathological basis [1]. Other ocular conditions are hypothesized to have a contributing vascular element to their pathogenesis e.g. glaucomatous optic neuropathy (GON) [2]. This necessitated the assimilation of various vascular imaging modalities in the management of such conditions. Vascular imaging modalities utilized in ophthalmology include among others dye-based angiographies e.g. fundus fluorescein angiography (FFA), Indocyanine green angiography (ICGA), color doppler imaging (CDI), laser doppler flowgraphy (LDF), laser speckle flowgraphy (LSF), retinal image analyzers (RIA) and optical coherence tomography angiography (OCTA) [3]. These modalities are complementary rather than interchangeable since each has its own advantages and limitations. OCT is one of the recent imaging modalities introduced to the field of ophthalmology. It has the advantage of providing in vivo microscopic-resolution 3D volumetric images than can be segmented into 2D slabs enabling isolation of selected tissue layers at a very high axial resolution. Various OCT machines have been introduced with different interferometry techniques and data processing algorithms including time-domain OCT (TD-OCT), spectral/Fourier domain OCT (SD-OCT), swept source OCT (SS-OCT) [4]. Faster image acquisition and higher scan rates enabled effective measurement of phase and amplitude fluctuations needed to detect motion within the sampled tissue which is the basis of OCTA [5]. While OCTA provides high spatial resolution of different vascular plexuses, it only indirectly derives velocimetric flow data through different methods and algorithms [6, 7]. Methods for deriving velocimetric flow data in OCTA include plane doppler OCT, phase-based velocimetry and intensity-based velocimetry like the split spectrum amplitude decorrelation angiography algorithm (SSADA). Each method has, however, its limitations. Phase-based and intensity-based algorithms are amenable to signal saturation dictated by a predetermined range of detectable phase difference and a finite temporal resolution of the OCT machine [6, 8]. In this study, we test the validity of a novel mathematical method aimed at calculating flow indices of the macular and ONH vasculature. This method profits from the high spatial resolution of OCTA while at the same time compensating for its velocimetry limitations. The law of Hagen-Poiseuille which quantitatively describes flow within cylindrical vessels has been extensively utilized as a model for the cardiovascular system physiology [9]. Our mathematical method combines the law of Hagen-Poiseuille, the vascular density (VD) measurements of OCTA and MOPP to generate reliable and repeatable flow indices that may compensate for OCTA velocimetric limitations e.g. saturation.

## Methods

This is a prospective study conducted at the ophthalmology department of Fayoum University Hospitals (FUH), was approved by the ethical committee of the faculty of medicine Fayoum University (IRB M488) and proceeded in compliance with the declaration of Helsinki. An informed consent was obtained from each participant. Inclusion criteria were a confirmed diagnosis of POAG according to the European Glaucoma Society Guidelines [10]. Exclusion criteria comprised angle closure glaucoma, secondary open angle glaucoma, previous glaucoma surgery, ocular occlusive vasculopathies e.g. retinal vein or artery occlusions, diabetic retinopathy, retinal vasculitides, non-glaucomatous optic neuropathies, retinal dystrophies and systemic vascular diseases with ocular complications e.g. diabetes mellitus, systemic vasculitides. Also, media opacities and low best corrected visual acuity (BCVA < 1/60), that would result in poor image quality index of angiograms, were among the exclusion criteria. Only angiograms of image quality index (IQI) of 5 or more were included. In this study, twenty-three Caucasian POAG patients were included. At baseline, fifteen patients had been on the fixed combination dorzolamide-timolol b.i.d. and the other eight on the prostaglandin analogue travoprost single nightly dose for at least three months prior to presentation. Four patients were hypertensive with no signs of hypertensive retinopathy and had been on bisoprolol for at least two years prior to recruitment. One male patient had been on tamsulosin for benign prostatic hyperplasia for three years prior to recruitment. Each participant underwent baseline assessment and a follow-up assessment one month later. During this intervening month, participants were prescribed brimonidine tartrate 0.2% b.i.d. for better IOP management since all had suboptimal IOP control at time of presentation. The assessment comprised full ophthalmological examination including BCVA, slit lamp anterior segment examination, gonioscopy, fundus examination. Also, systemic arterial blood pressure (SABP) both systolic (SBP) and diastolic (DBP) were measured using a pneumatic sphygmomanometer in the left brachial artery in a seated position with the back supported. In addition, IOP was measured in both eyes using the Perkins applanation tonometer (PAT) also in a seated position with the back supported. OCTA macula 6*6 mm and ONH 4.5*4.5 mm of both eyes were performed for each participant in a comfortable seated position by the same experienced operator. Baseline and follow-up assessments as well as imaging were done at a fixed time around 2:00 pm in order to avoid the effect of circadian fluctuations of IOP and SABP. Also, participants were instructed to abstain from smoking, caffeinated and alcoholic beverages for at least 12 h prior to assessment.

To test the validity of our mathematical flow indices (FIMs), the OCTA angiograms generated by the intensity-based algorithm SSADA of the AngioVue software version 2017.1.0.151 of the OCT apparatus (XR Avanti Optovue, Inc., Fremont, CA, USA) were used to derive OCTA intensity-based flow indices (FIOs) with the help of the image processing software ImageJ Version 1.2.4 software (RRID: SCR_003070). FIOs were then statistically correlated to their respective FIMs using Pearson correlation. Moreover, intraclass correlation coefficients (ICCs) were tested for each of the different indices in order to evaluate their repeatability and consistency between baseline and follow-up.

**Fig. 1:**
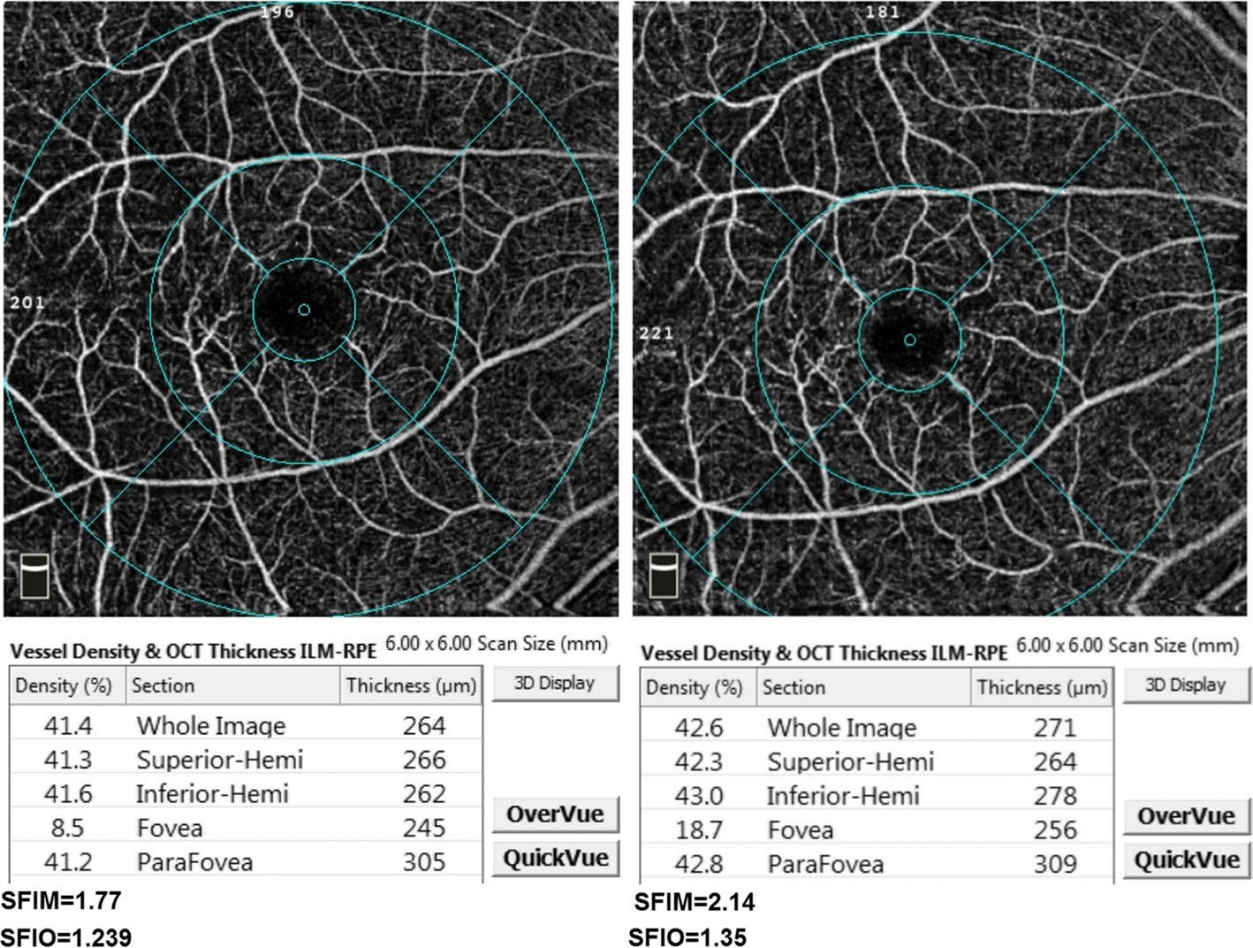
The superficial vascular plexus slab with superficial vascular density measurements for whole image, superior-hemi, inferior-hemi, fovea and parafovea at baseline (left) and follow-up (right) of one of the participants. The mathematical and OCTA intensity-based flow indices (SFIM and SFIO) are shown below their respective angiograms

**Fig. 2:**
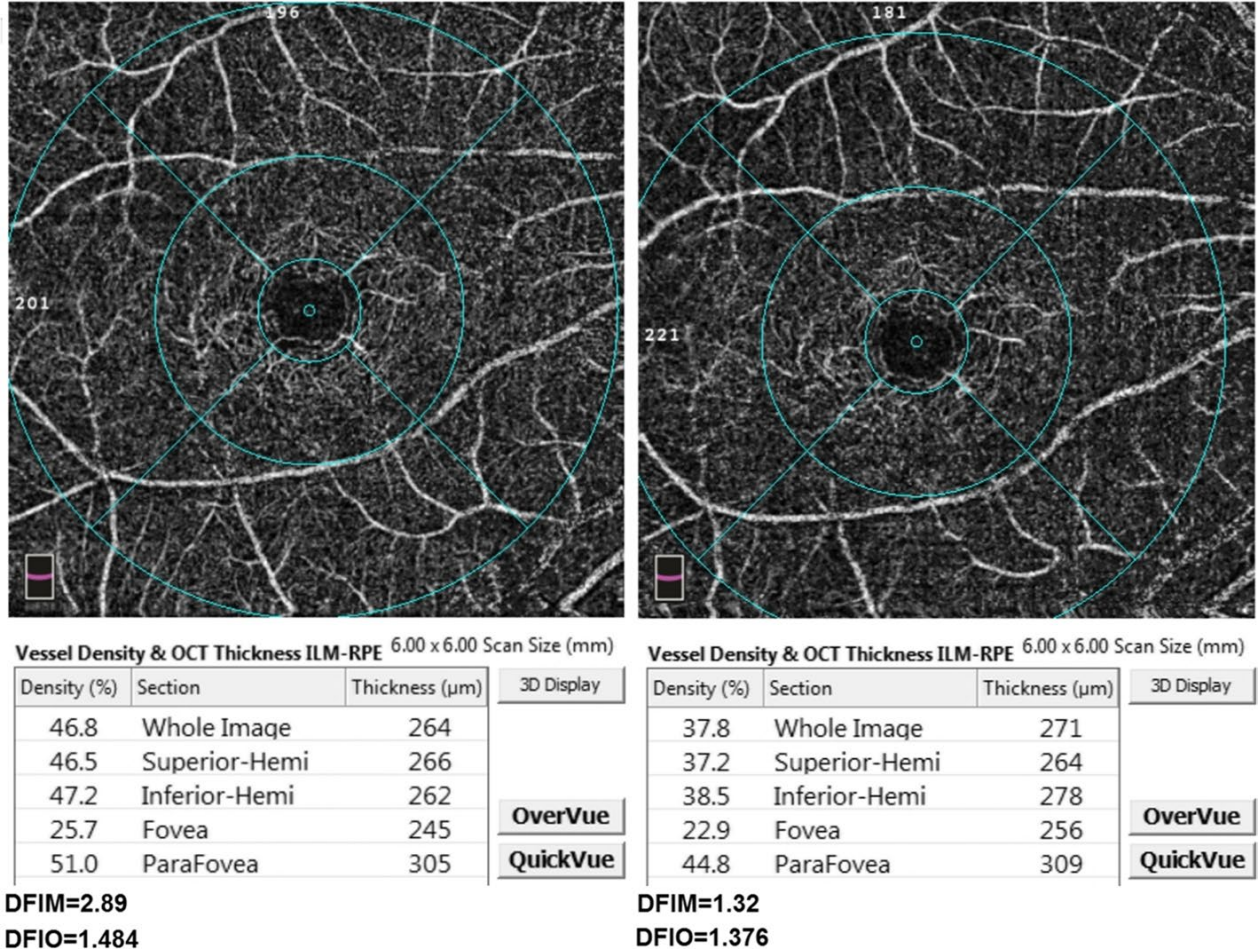
The deep vascular plexus slab with deep vascular density measurements for whole image, superior-hemi, inferior-hemi, fovea and parafovea at baseline (left) and follow-up (right) of one of the participants. The mathematical and OCTA intensity-based flow indices (DFIM and DFIO) are shown below their respective angiograms

**Fig. 3:**
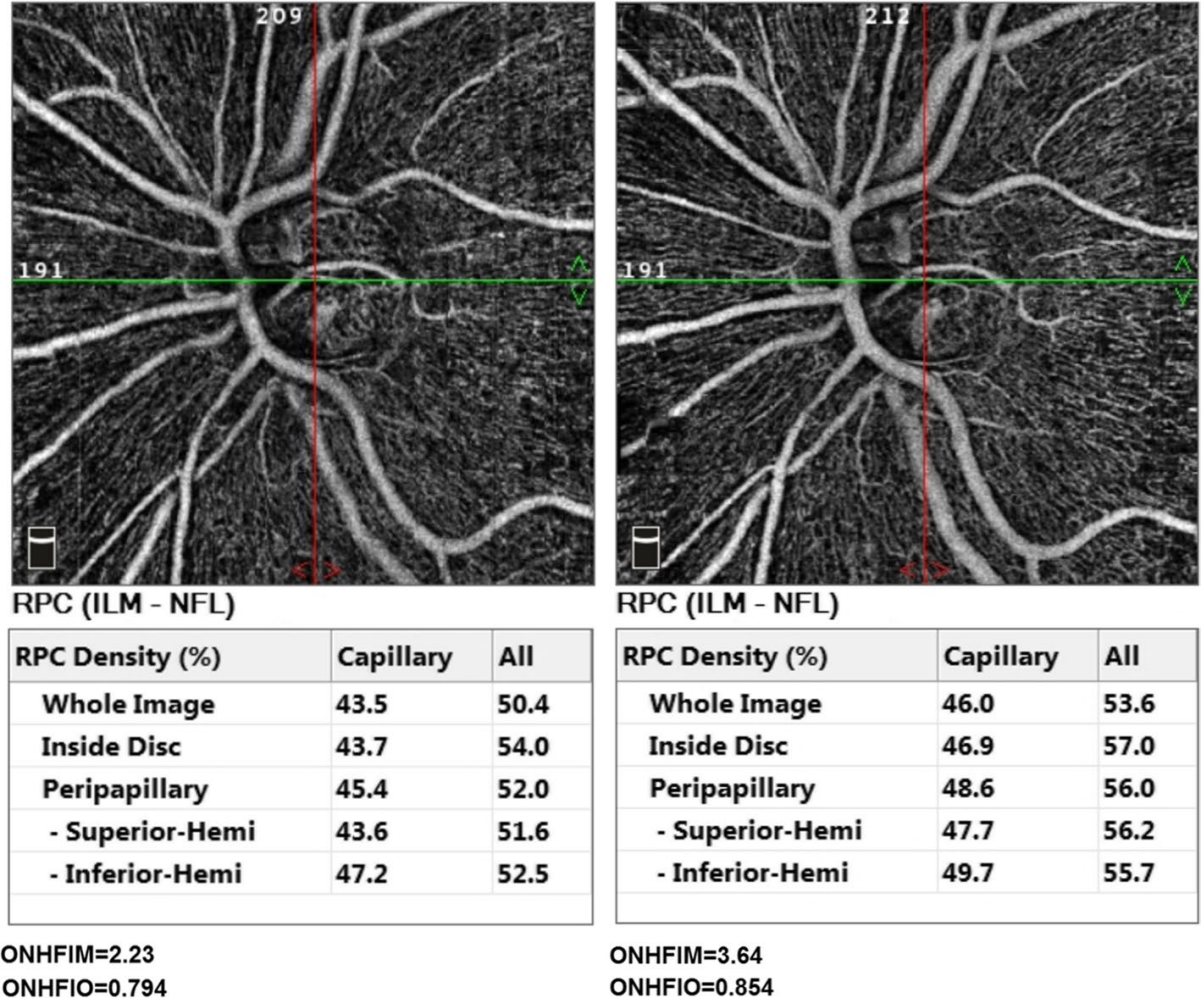
The optic nerve head (ONH) slab with ONH vascular density (ONHVD) measurements for whole image, inside disc, superior-hemi, inferior-hemi at baseline (left) and follow-up (right) of one of the participants. The mathematical and OCTA intensity-based flow indices (ONHFIM and ONHFIO) are shown below their respective angiograms

To calculate the FIOs, the OCTA angiograms 6*6 mm macula and 4.5*4.5 mm ONH were exported in JPEG format 1596*990 pixels. Only angiograms of image quality index 5 or more were included and a maximum image quality index difference of 1 was allowed between baseline and follow-up angiograms. The region of interest (ROI) tool of ImageJ was used to isolate the target 2D angiography slabs. The 2D slabs of interest in our study were the superficial vascular plexus (SVP), deep vascular plexus (DVP) and optic nerve head vascular plexus (ONH-RPC). The integrated density tool of ImageJ was then utilized to measure the integrated pixel density of each of the isolated slabs, SVPID, DVPID, ONHID for the SVP, DVP and ONH-RPC respectively. The integrated density measurement is the sum of all individual pixel densities in the 2D angiogram which is assumed to quantitatively relate to the total velocimetric flow in the angiogram. This is based on the aforementioned principle of the intensity-based SSADA where higher pixel density represents higher velocimetric flow [6]. The integrated density measurements were then multiplied by a fixed constant (C) to achieve a practical numerical range of the indices. The three calculated OCTA intensity-based flow indices were the SFIO, DFIO and ONHFIO for the SVP, DVP and ONH-RPC respectively Figs. 1, 2 and 3. The following equations summarize the calculation of FIOs:

$${\varvec{S}}{\varvec{F}}{\varvec{I}}{\varvec{O}}={\varvec{C}}*SVPID$$

$${\varvec{D}}{\varvec{F}}{\varvec{I}}{\varvec{O}}={\varvec{C}}*DVPID$$

$${\varvec{O}}{\varvec{N}}{\varvec{H}}{\varvec{F}}{\varvec{I}}{\varvec{O}}={\varvec{C}}*ONHID$$

$${\varvec{C}}={10}^{-7}$$

**Fig. 4:**
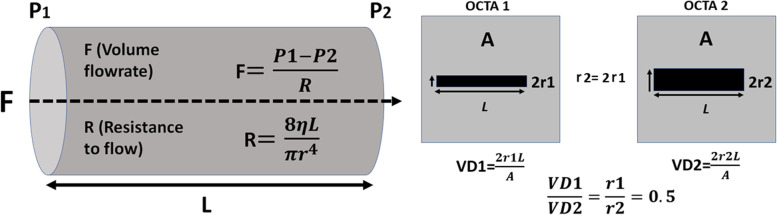
Diagrammatic illustration of Hagen-Poiseuille law (left) and the basis for using VD (vascular density) as surrogate for r (average vessel radius) (right); F (volumetric flow), P_1_ and P_2_ (perfusion pressure at the beginning and end of the vessel segment respectively), R (resistance to flow), L (total vessel length), ƞ (fluid viscosity). The grey square represents the total OCT scan area (A) and the black rectangle represent the total vessel area within the scan area

For the FIMs, we used the Hagen-Poiseuille’s flow equation ${\varvec{Q}}=\frac{{\varvec{\pi}}{\varvec{P}}{{\varvec{r}}}^{4}}{8\upeta {\varvec{L}}}$ where ***Q*** is the volumetric flow within a cylindrical vessel, ***P*** the perfusion pressure, ***r*** the radius of the vessel, ***ƞ*** fluid viscosity and ***L*** vessel length. The law of Hagen-Poiseuille (H-P) states that ***Q*** within a cylindrical vessel of uniform ***r*** is directly proportional to ***P*** and inversely proportional to flow resistance. The flow resistance is directly proportional to ***ƞ***, ***L*** and inversely proportional to ${\mathbf{r}}^{4}$ Fig. 4. In our study, ***P*** was assumed to be equal to MOPP which was calculated from SABP and IOP [11]. OCTA-measured vascular densities, the superficial vascular density (SVD), deep vascular density (DVD), optic nerve head vascular density (ONHVD) of the SVP, DVP and ONH-RPC respectively were used as surrogate for ***r*** in H-P equation. This is based on the fact that VD is the percentage area of blood vessels relative to the whole imaged area. Since the imaged area and the total vessel length for the same patient are anatomically predetermined by the imaging protocol e.g. 6*6 mm macula, 4.5*4.5 mm ONH, therefore, any change in VD for the same patient from baseline to follow-up is fully attributable to changes in average vessel radius, provided no vascular dropout has occurred, in the imaged area according to the simplified equation ${\varvec{V}}{\varvec{D}}=\frac{\mathbf{L}\mathbf{*}2\mathbf{r}}{\mathbf{A}}\mathbf{\%}$ where ***L*** is the total vessel length in the imaged area, ***r*** is the average vessel radius and ***A*** is the imaged area Fig. 4. The other variables in H-P equation ***ƞ*** and ***L*** were assumed to be constant for each patient at both baseline and follow-up. Consequently, the H-P flow equation in our mathematical method can be simplified as follows ${\varvec{Q}}={\varvec{E}}\boldsymbol{*}{\varvec{M}}{\varvec{O}}{\varvec{P}}{\varvec{P}}\boldsymbol{*}{{\varvec{V}}{\varvec{D}}}^{4}$. ***E*** is a constant of ${10}^{-8}$ to negate the percentage element of VD when subjected to the fourth power which achieves a practical numerical range of the index. The simplified H-P equation was applied on the SVP, DVP, ONH-RPC to calculate the respective FIMs i.e. SFIM, DFIM, ONHFIM. The following equations summarize the calculation of FIMs:

$${\varvec{M}}{\varvec{A}}{\varvec{B}}{\varvec{P}}\left(Mean\,Arterial\,Blood\,Pressure\right)=DBP+\frac{1}{3}\left(SBP-DBP\right)$$

$${\varvec{M}}{\varvec{O}}{\varvec{A}}{\varvec{P}}\left(Mean\,Ophthalmic\,Artery\,Pressure\right)=\frac{2}{3} MABP$$

$${\varvec{M}}{\varvec{O}}{\varvec{P}}{\varvec{P}}=MOAP-IOP$$

$${\varvec{S}}{\varvec{F}}{\varvec{I}}{\varvec{M}}={\varvec{E}}*MOPP*{SVD}^{4}$$

$${\varvec{D}}{\varvec{F}}{\varvec{I}}{\varvec{M}}={\varvec{E}}*MOPP*{DVD}^{4}$$

$${\varvec{O}}{\varvec{N}}{\varvec{H}}{\varvec{F}}{\varvec{I}}{\varvec{M}}={\varvec{E}}*MOPP*{ONHVD}^{4}$$

$${\varvec{E}}={10}^{-8}$$

MOAP is assumed to equal two thirds of MABP in a seated position because of the higher level of ophthalmic compared to brachial artery. Also, IOP is used as surrogate for ocular venous pressure [11]. Both FIOs and FIMs were calculated for each patient at baseline and follow-up. Statistical analysis was then performed to detect changes in these indices from baseline to follow-up and to determine the type and degree of correlation between the two sets using Pearson correlation. Also, ICCs were tested for baseline and follow-up values for each index.

## Statistical analysis plan

Descriptive statistics were presented in the form of numbers and percentages for categorical variables, mean and SD for numerical variables. Paired-t test was done to compare different measures between baseline and follow-up. Pearson correlation was done to show the correlation between FIOs and FIMs. ICCs were done for the baseline and follow-up values for each index. IBM SPSS 28 for windows software was used for analysis, and a P-value < 0.05 was considered statistically significant.

## Results

**Fig. 5:**
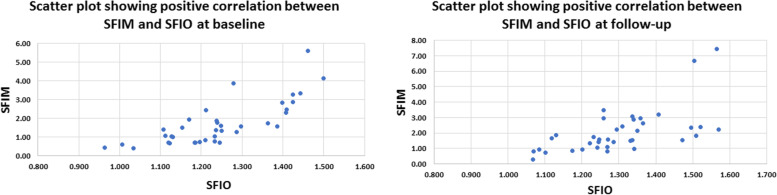
Scatter plot showing positive correlation between mathematical flow index of the superficial vascular plexus (SVP) (SFIM) and the OCTA intensity-based flow index of SVP (SFIO) at baseline (left) and follow-up (right)

**Fig. 6:**
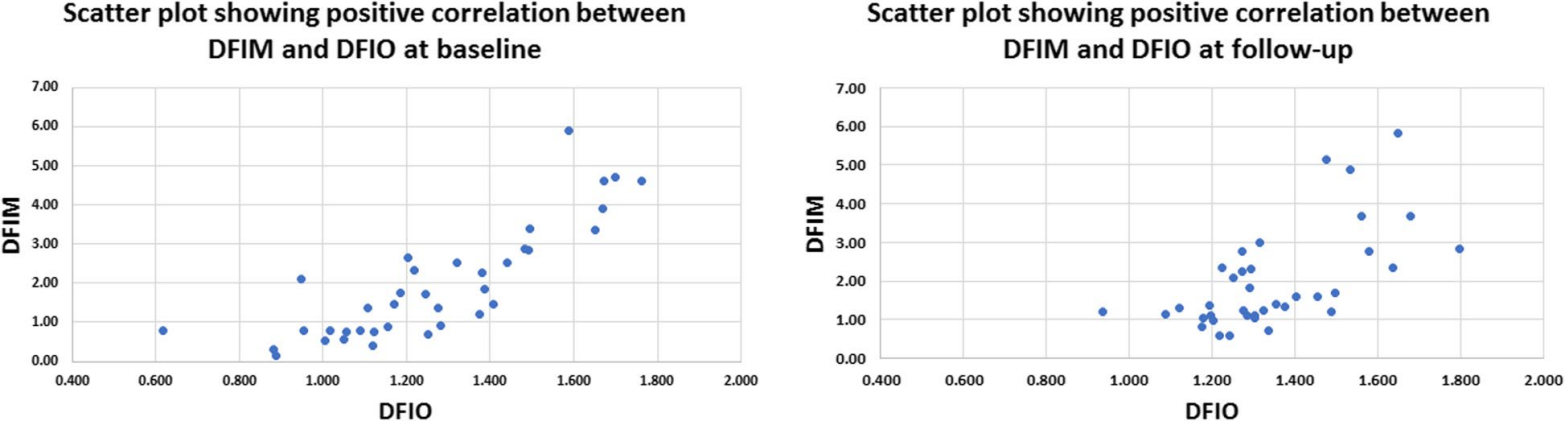
Scatter plot showing positive correlation between mathematical flow index of the deep vascular plexus (DVP) (DFIM) and the OCTA intensity-based flow index of DVP (DFIO) at baseline (left) and follow-up (right)

**Fig. 7:**
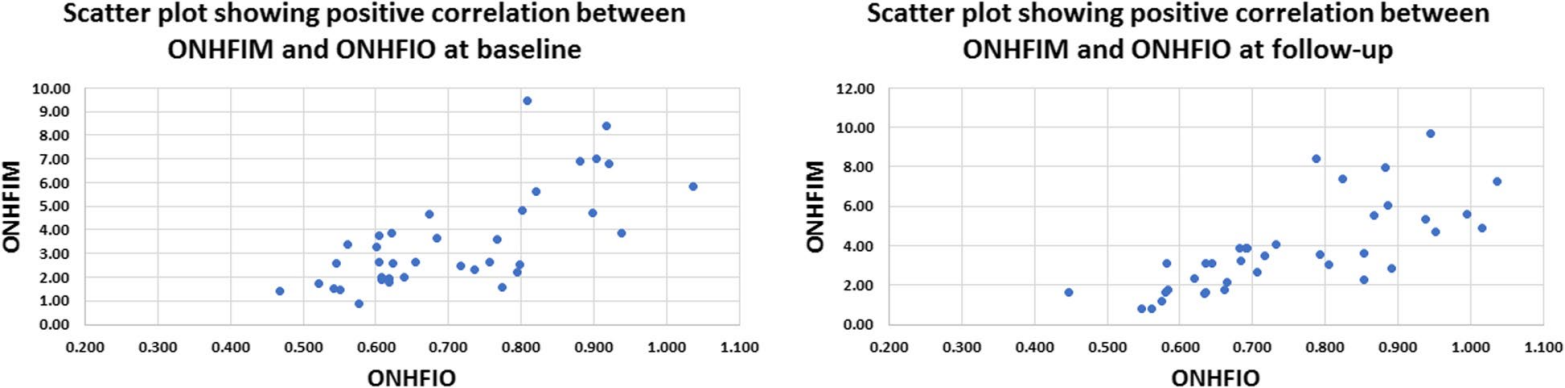
Scatter plot showing positive correlation between mathematical flow index of the optic nerve head vascular plexus (ONH-RPC) (ONHFIM) and the OCTA intensity-based flow index of ONH-RPC (ONHFIO) at baseline (left) and follow-up (right)

A total of 23 patients participated in this study of whom 61% were males. The mean and standard deviation (SD), median and interquartile range (IQR) of age were (56.7 ± 12.49) and (61.5, 50.75–65) years respectively. Data of thirty-seven eyes were analyzed at baseline and follow-up. Nine eyes were excluded because of image quality index below 5 due to significant media opacity or low visual acuity, poor fixation and profound motion artifacts. Twenty-two eyes (59.46%), eight eyes (21.62%) and seven eyes (18.92%) demonstrated mild, moderate and severe glaucomatous optic neuropathy according to the OCT retinal nerve fiber layer thickness (RNFLT)-based structural glaucoma damage staging [12]. Mean average RNFLT was 92.78 ± 20.89, 107.45 ± 11.24, 79.38 ± 0.16 and 62 ± 4.73μm for all eyes, eyes with mild, moderate and severe structural glaucomatous damage respectively Table 1. Detailed RNFLT measurements for different ONH quadrants are presented in Table 1. Mean Cup/Disc (C/D) area ratio was 0.29 ± 0.18, 0.21 ± 0.13, 0.45 ± 0.16 and 0.37 ± 0.22; mean C/D ratio vertical was 0.55 ± 0.19, 0.48 ± 0.17, 0.70 ± 0.11 and 0.61 ± 0.21; mean C/D ratio horizontal was 0.47 ± 0.18, 0.40 ± 0.15, 0.62 ± 0.13 and 0.55 ± 0.20 for all eyes and eyes with mild, moderate and severe glaucomatous damage respectively Table 1. All three FIMs i.e. SFIM, DFIM, ONHFIM demonstrated positive correlation with their respective FIOs i.e. SFIO, DFIO, ONHFIO. The Correlation coefficient (CC) for SFIM and SFIO was 0.773 *P*-value < 0.001 at baseline and 0.609 *P*-value < 0.001 at follow-up indicating high and moderate positive correlation respectively. Similarly, the DFIM and DFIO showed high and moderate positive correlation at baseline and follow-up respectively with CC of 0.829 *P*-value < 0.001 and 0.624 *P*-value < 0.001 respectively. Moreover, the ONHFIM and ONHFIO demonstrated moderate and high positive correlation at baseline and follow-up respectively with CC of 0.516 *P*-value = 0.001 and 0.734 *P*-value < 0.001 respectively Figs. 5, 6, 7 and Table 2

**Table 1:** Characteristics of participants

**Sex (N, %)**							
Male	14(60.8%)						
Female	9(39.13%)						
**Age (mean ± SD) (median, IQR)**	(56.7 ± 12.49) (61.5, 50.75–65)						
**No of eyes**	37						
No of eye according to structural glaucoma severity staging^*^ (N, %)							
Mild (RNFLT > = 85µm)	22(59.46%)						
Moderate (RNFLT > 70 and < 85µm)	8(21.62%)						
Severe (RNFLT < = 70µm)	7(18.92%)						
**RNFLT (mean ± SD)**	**Avg**	**Sup. H**	**Inf. H**	**I**	**S**	**N**	**T**
All	92.78 ± 20.89	93.41 ± 18.86	92.30 ± 24.32	112.05 ± 33.41	107.76 ± 25.37	82.22 ± 22.70	71.59 ± 12.19
Mild	107.45 ± 11.24	105.95 ± 10.95	109.09 ± 13.72	134.27 ± 18.03	122.86 ± 17.29	97.41 ± 13.58	77.14 ± 9.79
Moderate	79.38 ± 4.14	83.13 ± 7.57	76.50 ± 6.12	94.88 ± 14.05	94.38 ± 9.12	65.50 ± 8.72	66.63 ± 13.23
Severe	62.00 ± 4.73	65.71 ± 5.91	57.57 ± 8.54	61.86 ± 13.66	75.57 ± 20.82	53.57 ± 13.45	59.86 ± 6.79
**Cup/Disc Ratios (C/D) (mean ± SD)**		**C/D area**		**C/D vertical**		**C/D horizontal**	
All		0.29 ± 0.18		0.55 ± 0.19		0.47 ± 0.18	
Mild		0.21 ± 0.13		0.48 ± 0.17		0.40 ± 0.15	
Moderate		0.45 ± 0.16		0.70 ± 0.11		0.62 ± 0.13	
Severe		0.37 ± 0.22		0.61 ± 0.21		0.55 ± 0.20	

*Abbreviations: SD* Standard deviation, *IQR* Interquartile range, *RNFLT* Retinal nerve fiber layer thickness, *Avg.* Average, *Sup. H.* Superior hemi, *Inf. H.* Inferior hemi, *I* Inferior quadrant, *S* Superior quadrant, *N* Nasal quadrant, *T* Temporal quadrant, *C/D* Cup/disc ratio

**Table 2:** Bivariate correlation between the mathematical (FIMs) and OCTA intensity-based (FIOs) flow indices at baseline and follow-up

		**Pearson correlation**^a^	*P*-value*	**95% C.I**	
				**Upper**	**Lower**
**At baseline**					
**SFIM**	**SFIO**	0.773	** < .001**	0.598	0.877
**DFIM**	**DFIO**	0.829	** < .001**	0.690	0.909
**ONHFIM**	**ONHFIO**	0.516	**0.001**	0.231	0.720
**At follow up**					
**SFIM**	**SFIO**	0.609	** < .001**	0.356	0.780
**DFIM**	**DFIO**	0.624	** < .001**	0.376	0.789
**ONHFIM**	**ONHFIO**	0.734	** < .001**	0.539	0.855

*Abbreviations:* SFIM, DFIM, ONHFIM the mathematical flow indices for superficial vascular plexus (SVP), deep vascular plexus (DVP) and optic nerve head vascular plexus (ONH-RPC) respectively; SFIO, DFIO, ONHFIO the OCTA intensity-based flow indices for SVP, DVP, ONH-RPC respectively, *C.I.* (confidence interval)

^a^Pearson correlation was used to test the type and degree of correlation between FIMs and FIOs at baseline and follow-up

^*^Bold = statistically significant *P*-value < 0.05

**Fig. 8:**
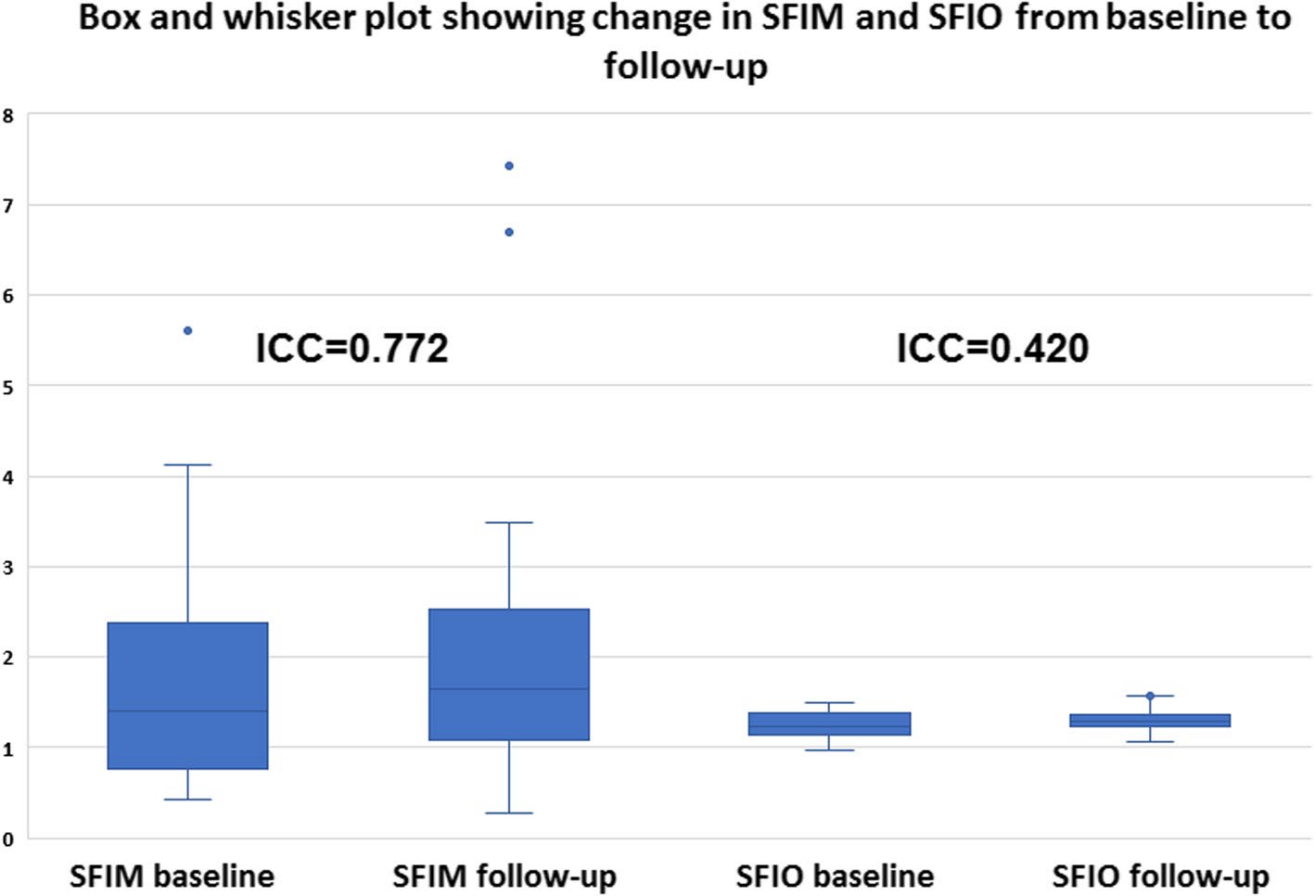
Box and whisker plot showing changes in mathematical flow index of the superficial vascular plexus (SVP) (SFIM) (left) and OCTA intensity-based flow index of the SVP (SFIO) (right) from baseline to follow-up; ICC (intraclass correlation coefficient)

**Fig. 9:**
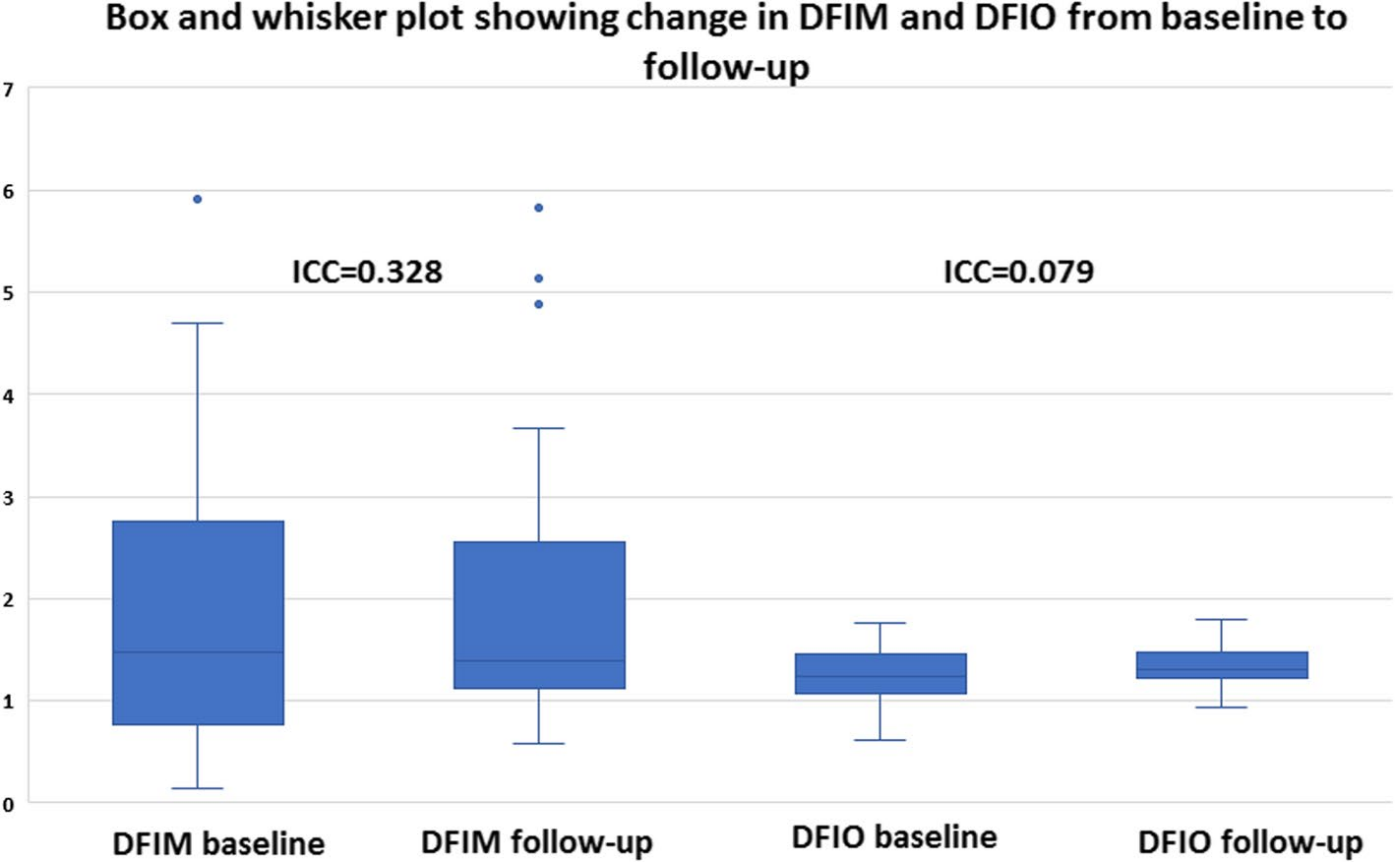
Box and whisker plot showing changes in mathematical flow index of the deep vascular plexus (DVP) (DFIM) (left) and OCTA intensity-based flow index of the DVP (DFIO) (right) from baseline to follow-up; ICC (intraclass correlation coefficient)

**Fig. 10:**
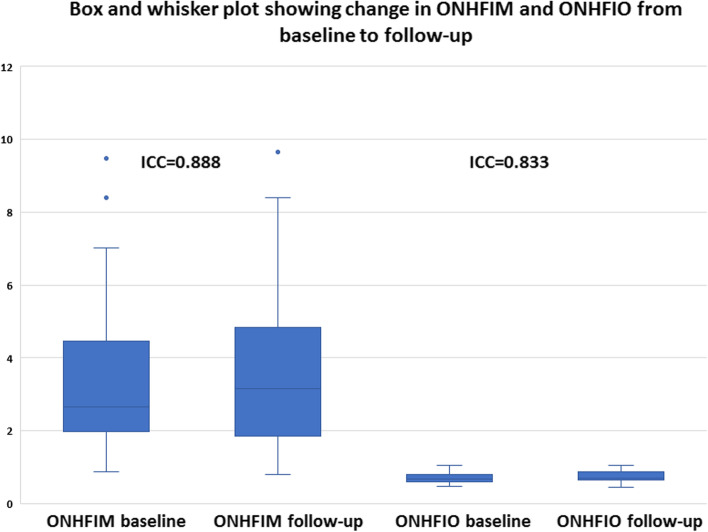
Box and whisker plot showing changes in mathematical flow index of the optic nerve head vascular plexus (ONH-RPC) (ONHFIM) (left) and OCTA intensity-based flow index of the ONH-RPC (ONHFIO) (right) from baseline to follow-up; ICC (intraclass correlation coefficient)

There was an increase in SFIM and SFIO from (M = 1.72, SD = 1.17) to (M = 2.07, SD = 1.45) *P*-value = 0.024 and from (M = 1.24, SD = 0.13) to (M = 1.30, SD = 0.14) *P*-value = 0.022 respectively at follow-up compared to baseline. Furthermore, ONHFIM and ONHFIO increased from (M = 3.53, SD = 2.09) to (M = 3.77, SD = 2.22) *P*-value = 0.153 and from (M = 0.70, SD = 0.14) to (M = 0.74, SD = 0.15) *P*-value = 0.012 respectively. In addition, the SVD for the whole image, superior-hemi and fovea increased from (M = 41.75, SD = 5.65) to (M = 43.51, SD = 5.41) P-value = 0.030, from (M = 42.07, SD = 5.47) to (M = 43.59, SD = 5.04) *P*-value = 0.002 and from (M = 15.55, SD = 8.04) to (M = 17.49, SD = 8.92) *P*-value = 0.030 respectively. Also, the ONHVD for inferior-hemi decreased from (M = 46.55, SD = 7.77) to (M = 45.32, SD = 8.16) *P*-value = 0.008. These statistically significant changes in SFIO, SFIM, SVD and ONHVD were attributed to brimonidine. No statistically significant changes could be demonstrated for BCVA LogMAR, IOP, SBP, DBP, MOPP, DVD, DFIM, DFIO Figs. 1, 2, 3 and Table 3. The intraclass correlation coefficients (ICCs) were 0.772 *P*-value < 0.001, 0.328 *P*-value = 0.022, 0.888 *P*-value < 0.001 for SFIM, DFIM and ONHFIM respectively. Also, ICCs for SFIO, DFIO and ONHFIO were 0.420 *P*-value = 0.004, 0.079 *P*-value = 0.320 and 0.833 *P*-value < 0.001 respectively Figs. 8, 9, 10 and Table 4.

**Table 3:** Comparison of different measures between baseline and follow-up expressed as mean and standard deviation (SD)

	**Baseline**	**After 1 month**	*P*-value*
	**Mean ± SD**	**Mean ± SD**	
**BCVA LogMAR**	0.56 ± 0.33	0.57 ± 0.33	0.172
**IOP**	17.97 ± 4.89	16.00 ± 5.76	0.064
**SBP**	145.00 ± 24.66	143.51 ± 37.02	0.724
**DBP**	80.41 ± 16.22	81.62 ± 19.30	0.664
**MOPP**	49.99 ± 13.36	52.17 ± 17.60	0.345
**SFIM**	1.72 ± 1.17	2.07 ± 1.45	**0.024**
**SFIO**	1.24 ± 0.13	1.30 ± 0.14	**0.022**
**DFIM**	1.94 ± 1.44	1.98 ± 1.28	0.902
**DFIO**	1.26 ± 0.26	1.35 ± 0.18	0.104
**ONHFIM**	3.53 ± 2.09	3.77 ± 2.22	0.153
**ONHFIO**	0.70 ± 0.14	0.74 ± 0.15	**0.012**
**SVD**			
Whole image	41.75 ± 5.65	43.51 ± 5.41	**0**.**030**
superior Hemi	42.07 ± 5.47	43.95 ± 5.04	**0.002**
Inferior Hemi	41.61 ± 6.42	42.95 ± 6.26	0.126
Fovea	15.55 ± 8.04	17.49 ± 8.92	**0.030**
Para fovea	42.60 ± 7.00	44.80 ± 5.61	0.068
Peri fovea	42.12 ± 6.01	43.79 ± 5.70	0.053
**DVD**			
Whole image	42.25 ± 7.58	43.24 ± 5.99	0.520
Superior Hemi	42.50 ± 7.61	43.95 ± 5.04	0.307
Inferior Hemi	42.26 ± 7.99	42.95 ± 6.26	0.622
Fovea	30.52 ± 9.98	30.55 ± 8.23	0.983
Para fovea	49.14 ± 6.40	49.84 ± 6.80	0.628
Peri fovea	42.24 ± 8.56	43.38 ± 6.90	0.521
**ONHVD**			
Whole image	50.38 ± 5.68	50.72 ± 6.16	0.451
RPCP	43.81 ± 5.59	43.88 ± 6.10	0.899
Inside Disc	44.31 ± 6.31	45.61 ± 6.11	0.119
Superior Hemi	46.84 ± 6.21	45.74 ± 7.01	0.160
Inferior Hemi	46.55 ± 7.77	45.32 ± 8.16	**0.008**
Inferior	48.49 ± 9.60	47.27 ± 8.89	0.195
Superior	46.32 ± 8.16	45.43 ± 8.62	0.243
Nasal	45.22 ± 10.79	44.00 ± 10.04	0.050
Temporal	46.05 ± 7.73	46.11 ± 9.60	0.955

*Abbreviations:* BCVA LogMAR, best corrected LogMAR visual acuity; IOP, intraocular pressure; MOPP, mean ocular perfusion pressure; SFIM, DFIM, ONHFIN, the mathematical flow indices for superficial vascular plexus (SVP), deep vascular plexus (DVP) and optic nerve head vascular plexus (ONH-RPC) respectively; SFIO, DFIO, ONHFIO, the OCTA intensity-based flow indices for SVP, DVP, ONH-RPC respectively

Paired-t test was done to show the change after 1 month for different measures

^*^Bold: statistically significant results *P*-value < 0.05

**Table 4:** Intraclass correlation coefficients (ICCs) for the baseline and follow-up values for each of the flow indices

	**Intraclass Correlation coefficient (ICC)**	**95% Confidence Interval (CI)**		*P*-value^*^
**SFIM**	0.772	0.601	0.876	** < 0.001**
**SFIO**	0.420	0.115	0.652	**0.004**
**DFIM**	0.328	0.009	0.587	**0.022**
**DFIO**	0.079	-0.248	0.389	0.320
**ONHFIM**	0.888	0.794	0.941	** < 0.001**
**ONHFIO**	0.833	0.696	0.911	** < 0.001**

*Abbreviations:* SFIM, DFIM, ONHFIM, the mathematical flow indices for superficial vascular plexus (SVP), deep vascular plexus (DVP) and optic nerve head vascular plexus (ONH-RPC) respectively; SFIO, DFIO, ONHFIO, the OCTA intensity-based flow indices for SVP, DVP, ONH-RPC respectively

^*^Bold = statistically significant *P*-value < 0.05

## Discussion

Various vascular mechanisms are major contributors to the pathogenesis of many ocular conditions e.g. vascular occlusion, leakage, hypoperfusion, vasospasm [2, 13–15]. Therefore, different vascular imaging modalities have been adopted in ophthalmology for the management and understanding of many ocular diseases [1]. The most commonly used modalities include dye-based angiographies i.e. FFA and ICGA, CDI, LDF, LSF and OCTA [3]. Among these imaging modalities, OCTA is noted for its high spatial resolution, 3D tissue sampling and 2D segmentation capabilities [16]. The introduction of SD-OCT and SS-OCT with faster scan rates and hence higher temporal resolution allowed for the development of OCTA which can construct a 3D image of the vascular plexuses within the sampled tissue based on its ability to detect areas of motion [16]. OCTA can only indirectly provide quantitative data about velocimetric flow unlike CDI which directly measures flow velocities. Various methods are used to derive velocimetric data from OCTA e.g. plane doppler OCT, phase-based OCT, intensity-based dynamic light scattering (iDLS-OCT) and SSADA [6]. Plane doppler OCT proved of little value in retinal imaging since the retinal blood flow is oriented nearly perpendicular to the incident OCT beam and therefore causes little doppler shift. Phase-based OCTA makes use of phase differences caused by moving particles within the sampled tissue, but is limited by a range of detectable phase differences determined by central OCT beam wavelength, tissue sampling frequency and doppler angle of the incident beam relative to the direction of motion. Therefore, velocities that create phase differences outside the predetermined range are undetectable [6]. On the other hand, the iDLS-OCT and SSADA are insensitive to perpendicular flow and rely on recording fluctuations in the OCT signal intensity which are dependent on the rate of flow of red blood cells, acting as optical scatterers, through the sampled tissue. However, the dependency of these methods on the rate and amplitude of signal fluctuations makes them liable to the saturation phenomenon where a very low or high flow results in signal fluctuations that are outside the resolvable temporal range of the OCTA machine [6, 8, 17]. In this study, we propose a novel mathematical method to calculate retinal and ONH flow indices that may help overcome the limitations of OCTA-based velocimetry. The proposed method profits from the high spatial resolution of OCTA by using the OCTA-measured VD as surrogate for average vessel radius. The MOPP and the VD, as a surrogate for average vessel radius, are used to calculate a flow index that is independent on velocimetric data from OCTA using the Hagen-Poiseuille flow equation which has been used in various models of the cardiovascular system physiology [9]. The calculation of FIMs requires no secondary image processing unlike FIOs which is a significant advantage. However, these mathematical indices have their own limitations. First, they assume a fixed quantitative relation between MABP and MOAP which may not stand under certain pathological conditions e.g. carotid insufficiency. Moreover, this method assumes that perfusion pressure i.e. MOPP is the same throughout the entire vascular bed including capillaries which is not accurate since the perfusion pressure progressively decreases from larger arterioles down to capillaries. However, retinal capillaries have a certain anatomical peculiarity that makes this approximation more acceptable. Retinal capillaries lack a well-defined precapillary sphincter which is thought to be responsible for a major drop of pressure from arterioles to capillaries and therefore, retinal capillary perfusion pressure is assumed to be higher than in capillaries elsewhere in the body e.g. limb capillaries [18]. Another disadvantage of this method is that it doesn’t differentiate between a decrease in VD secondary to vascular dropouts as opposed to a decrease in average vessel radius. Consequently, these FIMs are more suitable for longitudinal intra-patient follow-up rather than inter-patient comparison. Our results showed a positive correlation that ranged between high and moderate for the three FIMs with their corresponding FIOs. Additionally, our results showed that intraclass correlation coefficients (ICCs) were higher for the three mathematical indices FIMs than for their corresponding intensity-based indices FIOs which indicated better reliability. Moreover, ICCs were indicative of good reliability for SFIM and ONHFIM. However, ICC for DFIM was low, but still higher than DFIO. We suggest that low ICC for DFIM compared to SFIM and ONHFIM was due to the lack of statistically significant changes between baseline and follow-up values for DFIM unlike the statistically significant increase in both SFIM and ONHFIM Table 3. This was supported by the fact that despite low ICC, DFIM demonstrated the highest Pearson correlation with its respective DFIO compared to SFIM and ONHFIO Figs. 5, 6, 7, Table 2. This suggested that low ICC for DFIM reflected the lack of a statistically significant consistent change between baseline and follow-up rather than actual poor reliability. It is reported that retinal vascular autoregulation can maintain constant blood flow at a MOPP range that is 40% less or more than average normal MOPP [19]. Since the average MOPP in our study was around 50 mmHg, we can assume a range of retinal autoregulation between 30 and 70 mmHg Table 3. We can observe in the box and whisker plots that *outliers* were associated with high FIMs that were due to high MOPP exceeding the upper limit of retinal vascular autoregulation of 70 mmHg Figs. 8, 9, 10. In the box and whisker plots for SFIM, we can observe one outlier in the baseline visit and two outliers in the follow-up visit. The baseline outlier corresponded to a SFIM of 5.60 with a MOPP of 77 mmHg which exceeded the 70 mmHg limit of retinal vascular autoregulation. Similarly, the two follow-up outliers corresponded to SFIM of 6.69 and 7.42 with MOPP of 85 mmHg which also exceeded the 70 mmHg limit Fig. 8. Also, in the box and whisker plot of DFIM, one baseline and three follow-up outliers could be spotted with DFIM values of 5.91, 4,89, 5.83, 5,13 respectively with MOPP of 77, 85, 85 and 71 mmHg respectively which again all exceeded the 70 mmHg limit Fig. 9. Finally, the box and whisker plot of ONHFIM demonstrated two baseline and one follow-up outliers with ONHFIM values of 9.84, 8.40 and 9.66 respectively and concomitant MOPP of 77, 74 and 85 mmHg respectively clearly surpassing the 70 mmHg limit Fig. 10. It is noteworthy that despite these outliers for the three FIMs, none of their corresponding FIOs showed any outliers. We hypothesize that the absence of outliers for FIOs demonstrated the failure of the intensity-based SSADA to measure the high flow state resulting from MOPP exceeding the upper limit of retinal vascular autoregulation pressure of 70 mmHg due to the phenomenon of OCTA signal saturation.

## Conclusions

The novel FIMs are reliable alternatives to FIOs especially in the extremes of MOPP that can result in OCTA signal saturation. FIMs demonstrated higher ICCs than their corresponding FIOs. SFIM and ONHFIM showed high ICCs with excellent reliability. While DFIM demonstrated low ICC indicating poor reliability, it still performed better than its corresponding DFIO. FIMs are also much easier to calculate, requiring only three values i.e. VD, IOP and SABP, compared to FIOs which require, in many OCTA machines, sophisticated secondary image processing. However, they are more suitable for longitudinal intra-patient follow-up rather than inter-patient comparison and may be inaccurate in the presence of carotid insufficiency.

## Data Availability

Data is available from the corresponding author on reasonable request.

## Abbreviations

b.i.d: Bis in die (twice a day)
CC: Correlation coefficient
CDI: Color doppler imaging
DBP: Diastolic blood pressure
DFIM: Deep flow index mathematical
DFIO: Deep flow index OCTA intensity-based
DVD: Deep vascular density
DVP: Deep vascular plexus
DVPID: Deep vascular plexus integrated density
FFA: Fundus fluorescein angiography
FIMs: Mathematical flow indices
FIOs: OCTA intensity-based flow indices
GON: Glaucomatous optic neuropathy
H-P: Hagen-Poiseuille
ICC: Intraclass correlation coefficient
ICGA: Indocyanine green angiography
IOP: Intraocular pressure
LDF: Laser doppler flowgraphy
LSF: Laser speckle flowgraphy
MABP: Mean arterial blood pressure
MOAP: Mean ophthalmic artery pressure
MOPP: Mean ocular perfusion pressure
OCTA: Optical coherence tomography angiography
ONH: Optic nerve head
ONHFIM: Optic nerve head flow index mathematical
ONHFIO: Optic nerve head flow index OCTA intensity-based
ONHID: Optic nerve head integrated density
ONH-RPC: Optic nerve head-radial peripapillary capillary
ONHVD: Optic nerve head vascular density
PAT: Perkins applanation tonometer
POAG: Primary open angle glaucoma
RIA: Retinal image analyzer
ROI: Region of interest
SABP: Systemic arterial blood pressure
SBP: Systolic blood pressure
SD: Standard deviation
SD-OCT: Spectral domain optical coherence tomography
SFIM: Superficial flow index mathematical
SFIO: Superficial flow index OCTA intensity-based
SSADA: Split spectrum amplitude decorrelation algorithm
SS-OCT: Swept source optical coherence tomography
SVD: Superficial vascular density
SVP: Superficial vascular plexus
SVPID: Superficial vascular plexus integrated density
TD-OCT: Time domain optical coherence tomography
VD: Vascular density
